# The Influence of Lead-Related Venous Obstruction on the Complexity and Outcomes of Transvenous Lead Extraction

**DOI:** 10.3390/ijerph18189634

**Published:** 2021-09-13

**Authors:** Marek Czajkowski, Wojciech Jacheć, Anna Polewczyk, Jarosław Kosior, Dorota Nowosielecka, Łukasz Tułecki, Paweł Stefańczyk, Andrzej Kutarski

**Affiliations:** 1 Department of Cardiac Surgery, Medical University of Lublin Poland, 20-078 Lublin, Poland; mczajkowski@interia.pl; 2 2nd Department of Cardiology, Faculty of Medical Science in Zabrze, Medical University of Silesia, Zabrze, 40-055 Katowice, Poland; wjachec@interia.pl; 3 Department of Physiology, Pathophysiology and Clinical Immunology, Collegium Medicum of Jan Kochanowski University, 25-736 Kielce, Poland; 4 Department of Cardiac Surgery, Świętokrzyskie Cardiology Center, 25-736 Kielce, Poland; 5 Department of Cardiology, Masovian Specialistic Hospital, 26-617 Radom, Poland; jaroslaw.kosior@icloud.com; 6 Department of Cardiology, The Pope John Paul II Province Hospital, 22-400 Zamość, Poland; dornowos@wp.pl (D.N.); paolost@interia.pl (P.S.); 7 Department of Cardiac Surgery, The Pope John Paul II Province Hospital, 22-400 Zamość, Poland; luke27@poczta.onet.pl; 8 Department of Cardiology, Medical University of Lublin Poland, 20-078 Lublin, Poland; a_kutarski@yahoo.com

**Keywords:** lead-related venous obstruction, transvenous lead extraction, lead extraction complications, lead extraction complexity

## Abstract

Background: Little is known about lead-related venous stenosis/occlusion (LRVSO), and the influence of LRVSO on the complexity and outcomes of transvenous lead extraction (TLE) is debated in the literature. Methods: We performed a retrospective analysis of venograms from 2909 patients who underwent TLE between 2008 and 2021 at a high-volume center. Results: Advanced LRVSO was more common in elderly men with a high Charlson comorbidity index. Procedure duration, extraction of superfluous leads, occurrence of any technical difficulty, lead-to-lead binding, fracture of the lead being extracted, need to use alternative approach and lasso catheters or metal sheaths were found to be associated with LRVSO. The presence of LRVSO had no impact on the number of major complications including TLE-related tricuspid valve damage. The achievement of complete procedural or clinical success did not depend on the presence of LRVSO. Long-term mortality, in contrast to periprocedural and short-term mortality, was significantly worse in the groups with LRSVO. Conclusions: LRVSO can be considered as an additional TLE-related risk factor. The effect of LRVSO on major complications including periprocedural mortality and on short-term mortality has not been established. However, LRVSO has been associated with poor long-term survival.

## 1. Background

Permanent cardiac pacing remains the leading treatment for patients with various rhythm disorders, conduction disturbances and ventricular arrhythmias. In recent years, we have also observed an increase in the implantation of more complex devices used in the prevention of sudden cardiac death and in the treatment of severe heart failure. In spite of technological progress over the last decade, conventional pacemakers, implantable cardioverter-defibrillators and cardiac resynchronization therapy (PM/ICD/CRT) devices still have endocardial leads. However, after the beginning of the endocardial pacing era only a few studies have investigated lead-related venous stenosis/occlusion (LRVSO) [1,2,3,4,5,6,7,8,9,10,11,12,13,14,15,16,17,18,19,20]. Various lead-related problems (infectious and non-infectious) are an inherent component of permanent endocardial pacing, and transvenous lead extraction (TLE) is considered an essential technique in lead management strategy [21,22,23,24]. TLE is a complex procedure that sometimes may lead to fatal complications such as venous or cardiac injury. Carrying out the procedure is often associated with technical problems and requires additional approaches and tools [22,23,24,25]. There are numerous reports on the estimation of the real risk of the TLE [26,27,28,29] but none of them considered LRVSO as a predictor of procedure difficulties. Among twenty reports on LRVSO [1,2,3,4,5,6,7,8,9,10,11,12,13,14,15,16,17,18,19,20], only three papers analyzed the occurrence of LRVSO before TLE [2,6,14], and only two considered the influence of LRVSO on procedure complexity providing at the same time conflicting results. Among 20 reports on LRVSO only two studies were carried out in populations over 200 patients [1,2], 10 in 100–150 participants [3,4,5,6,7,8,9,10,11,12] and the remaining eight studies in populations consisting of 30–89 patients [13,14,15,16,17,18,19,20]. In this study, a total of 2909 TLE procedures were preceded by venography and LRVSO was documented in 2138 venograms. Ipsilateral venography before TLE is an integral part of the procedure (in the absence of contraindications for contrast intake).

### Goal of the Study

The aim of this study was to determine the incidence of varying degrees of LRVSO and to examine the influence of LRVSO on procedure difficulty, complexity, major complications related to TLE, procedure effectiveness as well as mid- and long-term mortality after TLE.

## 2. Methods

### 2.1. Study Population

A post-hoc analysis of clinical data from 2909 patients undergoing transvenous lead extraction (TLE) between June 2008 and March 2021 at a single high-volume center was performed. All information regarding the patients and the procedures were entered into the computer database on a current basis. Patients with medical contraindications for venography (contrast intake) were excluded from the study.

Table 1 summarizes the most important information regarding the study population.

The current study uses data from a high-volume center that performs more than 200 TLE per year. 

The percentage of serious complications is relatively higher compared to other reports, however, in the presented center, the most difficult procedures in the country are performed.

The first line tools used in study center are conventional mechanical sheaths, powered rotational mechanical sheaths and other instruments are second-line tools. Excimer laser sheaths are not used.

### 2.2. Venography

Preoperative venography was performed in 2909 patients submitted for transvenous lead extraction between June 2008 and March 2021 at our high-volume center. A peripheral intravenous catheter was placed in the peripheral arm vein on the side (or both sides of the chest) to be examined. All patients received an injection of 20–40 mL high-quality contrast medium (350 mg iodine/mL) Iomeron 350 into the peripheral arm vein on the side of endocardial lead implantation. Venous blood flow in the upper arm, neck and chest was recorded by cine-angiography. All images were acquired in the anteroposterior view. The venograms were obtained in a single plane (anterior–posterior) and stored on CD-ROM discs. An experienced cardiologist and experienced (trained by an interventional radiologist) cardiac surgeon reviewed the venograms, and venous patency was graded on a five-degree scale from normal flow to complete occlusion. All venograms were obtained in the same manner. Venographic analysis: at baseline, the narrowest and widest points of the target vessel for lead placement were identified by visual inspection to obtain minimum and maximum venous diameters, and measurements from two to three individually calibrated frames were averaged to determine the final status of the vein as no stenosis, mild stenosis (<50% narrowing), moderate stenosis (50–80% narrowing), severe stenosis (≥80% narrowing) and complete occlusion of the axillary (AxV), subclavian (ScV), innominate (brachiocephalic) (AnV) veins and superior vena cava (SVC). In spite of contrast injection in the arm vein on the side of the endocardial lead, regional collateral blood vessels and venous collateral blood flow in the neck enabled evaluation of the brachiocephalic vein on the opposite side of the chest. What is the significance of this classification of vessel narrowing in clinical practice? LVRSO was graded according to our own, arbitrarily estimated, criteria, which rely to the remaining effective vein lumen necessary for different electrodes/catheters safe passage.

Mild narrowing: possible insertion of a new/additional lead using standard introducers, central venous catheters, permanent catheters for hemodialysis and there is a chance that the arteriovenous (AV) fistula will work properly.

Moderate narrowing: probable insertion of a new lead but hydrophilic guide wires and longer introducers are necessary, possible insertion of central venous catheters (troubles possible), possible insertion of permanent catheters for hemodialysis and there is a small chance that the AV fistula will work properly.

Severe narrowing: impossible insertion of a new lead, hydrophilic guide wires and longer introducers might be helpful, insertion of central venous catheters may be risky, chances to pass a catheter for hemodialysis without venoplasty are very small and there is no chance that the AV fistula will work properly.

Complete occlusion: no chance to pass a hydrophilic guide wire; only lead extraction and regaining venous access enables the insertion of a new lead.

Reuse of occluded veins and technical aspects of lead extraction/replacement depend not only on maximal venous narrowing but also on the length of the narrowing (the number of the affected vessels, too).

### 2.3. Lead Extraction Procedure

Lead extraction procedures were defined according to the most recent guidelines on management of lead-related complications (HRS 2017 and EHRA 2018) [21,22,23]. Indications for TLE and type of periprocedural complications were defined according to the 2017 HRS Expert Consensus Statement on Cardiovascular Implantable Electronic Device Lead Management and Extraction [22].

All procedures were performed using non-powered mechanical systems such as polypropylene Byrd dilator sheaths (Cook^®^ Medical, Leechburg, PA, USA), mainly via the implant vein. If technical difficulties arose, alternative venous approaches and/or additional tools such as Evolution (Cook^®^ Medical, Leechburg, PA, USA), TightRail (Spectranetix, Colorado Springs, CO, USA), lassos, basket catheters were used. Excimer laser sheaths were not used.

All extraction procedures were performed following different organizational models spanning 25 years of experience. In the initial era of lead extraction, the procedures were performed in the electrophysiology laboratory using intravenous analgesia/sedation; then the recommended safety precautions were observed to perform more complex and risky procedures in the operating theater, and finally in the hybrid room under general anesthesia. The core extraction team has consisted of the same very experienced TLE operator and a dedicated cardiac surgeon with an experienced echocardiographist over the last six years.

### 2.4. TEE Monitoring during TLE

TTE, pre- and postoperative TEE were mandatory (excluding contraindications) from the very beginning. Continuous transesophageal echocardiographic (TEE) monitoring has been an important standard tool over the last six years [30,31,32]. TEE in our series was performed using Philips iE33 or GE Vivid S 70 machines equipped with X7-2t Live 3D or 6VT-D probes. All recordings were archived and consisted of pre-procedural examination, navigation of lead removal and post-procedural evaluation of the efficacy of the procedure with an assessment of possible complications [30,31,32]. The intra-procedural phase of TEE monitoring allowed visualization of pulling on the cardiac walls and invagination of the right ventricle during lead removal, followed by a drop in systolic blood pressure in response to this maneuver. Continuous monitoring made it possible to clarify the cause of blood pressure fall during TLE [30,31,32].

### 2.5. Statistical Analysis

The Shapiro–Wilk test showed that most continuous variables were normally distributed. For uniformity, all continuous variables are presented as the mean ± standard deviation. The categorical variables are presented as number and percentage. In the first step the Kruskal–Wallis ANOVA test was used to determine whether there were statistically significant differences between groups. Next, the variables achieving *p* < 0.1 were compared using the nonparametric Chi^2^ test with Yates correction (dichotomous data) or the unpaired Mann–Whitney U test (continuous data), as appropriate. Comparisons were made between Groups 1 and 2 vs. Groups 4 and 5. A *p*-value less than 0.05 was considered as statistically significant. In order to assess the effect of LRVSO on mortality, Kaplan–Meier survival curves were plotted, the course of which was assessed using the log rank test. Statistical analysis was performed with Statistica version 13.3 (TIBCO Software Inc., Palo Alto, CA, USA).

### 2.6. Approval of the Bioethics Committee

All patients gave their informed written consent to undergo TLE and use anonymous data from their medical records, approved by the Bioethics Committee at the Regional Chamber of Physicians in Lublin No. 288/2018/KB/VII. The study was carried out in accordance with the ethical standards of the 1964 Declaration of Helsinki.

## 3. Results

### Patient Groups

For the purposes of analysis, the study population was divided into five groups according to venogram results, namely Group 1—no stenosis (499 patients), 2—mild stenosis (574 pts), 3—moderate stenosis (605 pts), 4—severe stenosis (581 pts) and 5—total occlusion (650 pts). Only maximal venous narrowing was considered as a criterion in patient selection.

Table 2, Table 3 and Table 4 summarize specific patient-, system- and procedure-related risk factors for procedure complexity, efficacy, complications and long-term mortality after TLE.

Analysis of the clinical factors demonstrated that lead-related stenosis/occlusion correlated with patient age during TLE, male gender and Charlson comorbidity index. Other patient-related risk factors for major complications, i.e., indications for CIED implantation, functional NYHA class III and IV, decreased LVEF, renal failure and previous sternotomy were not related to LRVSO (Table 2).

Analysis of CIED systems and history of pacing showed that venous stenosis or lead-related (LR) total venous occlusion were more frequent in CRT-D recipients. Patients with ICD (VVI, DDD) were less likely, albeit insignificantly to have total venous occlusion.

Patients with redundant loops of the lead before TLE, leads with proximal end in the coronary sinus vein (CSV) and a higher number of CIED-related procedures before lead extraction were more likely to have severe venous stenosis or total occlusion.

Patients with severe venous stenosis or LR total venous occlusion had more risk factors for major complications (MC) and higher procedure complexity estimated with the SAFeTY TLE calculator [26]. These patients also had multiple leads to be removed (including three or more leads), they were more likely to require venous approach on both sides of the chest, extraction of leads with redundant loops in the heart, extraction of abandoned lead (s) and extraction of lead (s) with long or very long implant duration (Table 2). 

Analysis of TLE complexity and degree of LRVSO showed that procedure duration (sheath-to-sheath), extraction of non-functional superfluous leads, occurrence of any technical problem during TLE, lead-to-lead binding, lead fracture during extraction, need to change venous approach, coincidence of three or more technical problems and necessity of using metal sheaths and lasso catheters/snares were associated with the presence of LRVSO (Table 3).

The occurrence of any major complication, urgent rescue cardiac surgery, partial radiographic success (remained tip or <4 cm lead fragment), damage to chordae tendineae, other forms of TLE-related TV dysfunction/damage, complete clinical success and complete procedural success as well as procedure-related death (intra-, post-procedural) did not show any relationship with LRVSO, similar to mortality in the first day, first month and first year after TLE. In contrast, mortality at more than one-year follow-up was significantly higher among patients with severe venous stenosis and complete venous occlusion (Table 4).

**Figure 1:**
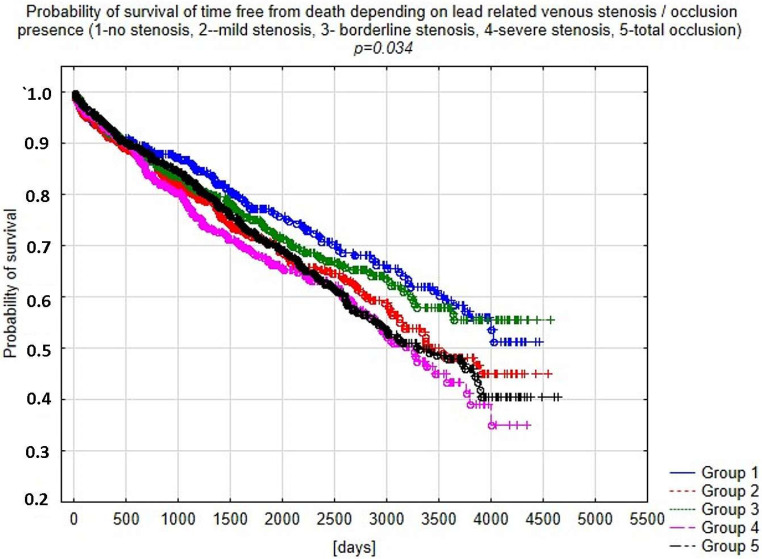
Probability of survival of patients undergoing TLE depending on the degree of LRVSO.

Analysis of mortality using the Kaplan–Meier curve confirmed the relationship between LRVSO and long-term survival after TLE (Figure 1).

## 4. Discussion

Venous obstruction is a well-known complication after implantation of a permanent transvenous pacemaker. The incidence of venous obstruction reaches 30–45% with complete occlusion rates of 12% on average and 1–3% for symptomatic occlusion [1,2,3,4,5,6,7,8,9,10,11,12,13,14,15,16,17,18,19,20]. In the current study, severe venous obstruction was identified in 19.94% (40.77% if moderate occlusion was included) whereas complete occlusion in 22.34% of patients. The higher incidence rate of total occlusion in the present study may be a result of long implant duration: cumulative dwell time of the extracted leads was 15.31 ± 12,925 years. Closer evaluation of the clinical factors showed that LVRSO was more common in elderly males with a higher Charlson comorbidity index. Several investigators confirm the contribution of various clinical factors to the occurrence of venous complications [4,5,6], others show no association between LRVSO and the clinical condition of the patient [7,11]. Analysis of the system/procedure-related factors in the present study demonstrated that the number of extracted leads, lead extraction on the left side or both sides of the chest, extraction of the lead with redundant loop in the heart, extraction of abandoned leads, extraction of leads with long implant duration and a higher risk of MC estimated using the SAFeTY TLE calculator [26] were related to the presence of severe venous stenosis or total venous occlusion. LRVSO was also more common in patients with CRT, having leads with their proximal end in the CVS and a higher number of CIED-related procedures before lead extraction. A similar relationship, especially between the number of extracted leads/long implant duration and LRVSO has been shown in previous reports [5,11].

Out of 20 reports, only four described LRVSO diagnosed just before the TLE procedure [2,6,13,14], and only two assessed the influence of LRVSO on the complexity of TLE [2,6]. The last two studies provide contradicting results. Li et al. in a study of 202 patients concluded that the presence of LRVSO made it more difficult to extract the leads, requiring advanced tools and more time [2]. In contrast, Boczar et al. in a group of 133 pts demonstrated that LRVSO did not influence the effectiveness, safety, and the use of additional tools during TLE procedures [6]. In the present study, the indicators of procedural difficulty and complexity such as procedure duration, extraction of superfluous leads, occurrence of any technical problem, lead-to-lead binding, fracture of the extracted lead, need to change venous approach, coincidence of three or more so-called technical problems and need to use metal sheaths or lasso catheters were related to the presence of LRVSO. The occurrence of any major complication was insignificantly higher in groups with LRVSO as compared to groups without significant stenosis: 2.754 and 2.461% vs. 1.603% and 1.742%, respectively. The need to perform urgent rescue cardiac surgery, partial radiographic success and damage to chordae tendinae during TLE were not significantly associated with the degree of LRVSO. The occurrence of TLE-related TV damage, achievement of complete clinical success and complete procedural success as well as procedure-related death (intra-, post-procedural) were unrelated to LRVSO, similar to mortality in the first day, first month and first year after TLE. This study, however demonstrated a link between TLE difficulty/complexity and the degree of LRVSO, which may be a reflection of implant duration and the total number of extracted leads. Thus, the real problem is only with implantation of new lead (s) because of lead dysfunction or necessity of upgrading the CIED system.

The pathophysiology of LRVSO is not well understood. It is likely that lead-related endothelial trauma incites an inflammatory response of the vessel wall with subsequent thrombosis and scarring. Early (days, weeks) LRVSO seems to be a result of thrombosis which can be treated with low-molecular heparin [16,17,18,19,20]. The role of thrombosis in delayed (months) or late (years) LRVSO is less clear. The inflammatory response of the vessel wall probably induces the formation of scar tissue similar to lead adhesion to the vessel and heart structures, observed on the extracted leads and during TEE [33]. The process of natural maturation makes lead-related fibrotic scar harder and harder leading to its mineralization and calcification. It is well-known that scar tissue in the SVC and in the heart makes lead dissection more difficult [34]. However, so far, nobody has considered scar tissue causing LRVSO and scar tissue around the leads detected during TEE/ICS as the same phenomenon. Looking at narrowing or occlusion of implant veins from this viewpoint we can explain the relationship between LRVSO and TLE complexity, difficulty and complications. Lead dissection in scarred veins is more effort-consuming and sometimes requires stronger pulling on the lead to be extracted. It can also explain the mechanism of TV damage during TLE (fortunately rare). It seems to confirm the concept of simultaneous lead traction from above and below during dissection; it can protect both the SVC wall and the TV [35]. Our results seem to confirm the significance of routine venography before TLE and considering LRVSO as still another risk factor for TLE complexity and major complications.

In the present study, worse long-term survival was demonstrated in patients with a higher degree of LRVSO. The reason for the worse survival rate in this group is not clear and is probably related to other factors as well (possibly a higher Charlson index).

## 5. Study Limitations

Our study has several limitations worth noting. Routine venography before TLE was performed in all patients except those with contraindications, mainly renal failure. For this reason, an interesting patient subpopulation had been excluded from the study. The database was prospectively integrated, but analysis was performed retrospectively. For the purposes of this study, the population of patients was divided into groups according to maximal venous narrowing without taking into account the site of narrowing/occlusion and the length of venous stenosis/occlusion. Therefore, the present analysis of venograms includes maximal venous narrowing but not the volume of the phenomenon (the number of vessels affected). The classification of patients we used in the study not only enabled comparison of our results with the findings of other investigators, but also maximal venous narrowing was considered a practical marker for predicting the usefulness of veins for implantation of a new lead/catheter.

## 6. Conclusions

The occurrence of significant venous stenosis/occlusion in patients undergoing TLE is related to some clinical factors (age, male gender, high Charlson comorbidity index) and numerous procedure-related factors, especially long implant duration, extraction of leads with redundant loop in the heart, extraction of abandoned leads, presence of leads with proximal end in the coronary sinus vein and a higher number of CIED-related procedures before lead extraction. LRVSO can be considered as an additional risk factor for TLE complexity. Further research is required to provide evidence for the relationship between scar tissue density encapsulating the leads visible in TEE and the degree of LRVSO. Lead-related venous stenosis/occlusion has no influence on mortality at one-year follow-up, but the presence of severe forms of LRVSO is associated with worse prognosis of patients undergoing TLE at more than one-year follow-up.

## Figures and Tables

**Table 1:** Characteristics of the study group.

All Patients (2909)	Mean/Number	SD/%
Patient age during TLE [years]	66.90	13.99
Patient age at first implantation [years]	58.51	15.67
Sex (% of female patients)	1147	39.43%
Etiology: IHD, MI	1676	57.61%
Etiology: cardiomyopathy, valvular heart disease	448	15.40%
Etiology: congenital, channelopathies, neurocardiogenic, post cardiac surgery	784	26.95%
LVEF [%]	48.89	15.21
Renal failure (any)	607	20.87%
Previous sternotomy	435	14.95%
Charlson comorbidity index [number of points]	4.775	3.625
Systemic infection (with pocket infection or not)	599	20.59%
Local (pocket) infection	253	8.70%
Lead failure (replacement)	1505	57.74%
Change of pacing mode/upgrading, downgrading	176	6.05%
Other (abandoned lead/prevention of abandonment (AF, superfluous leads), threatening/potentially threatening lead (loops, free ending, left heart, LDTD), other (MRI indications, cancer, painful pocket, loss of indications for pacing/ICD), regaining venous access (symptomatic occlusion, SVC syndrome, lead replacement/upgrading)	374	12.86%
System: pacemaker (any)	2013	69.20%
System: ICD-V, ICD-D	281	22.93%
System: CRT-D	667	7.80%
Dwell time of the oldest lead per patient before TLE [months]	101.5	75.57
Cumulative lead dwell time before TLE [years]	15.31	12.925
Major complications: all	61	2.10%
Major complications (with rescue cardiac surgery)	35	1.20%
Major complications (without rescue cardiac surgery)	7	1.20%
Minor complications	174	5.98%

AF—atrial fibrillation, CRT-D: implantable cardioverter-defibrillator with resynchronization function, ICD—implantable cardioverter-defibrillator, ICD-D—dual chamber implantable cardioverter-defibrillator, ICD-V—single chamber implantable cardioverter-defibrillator, IHD—ischemic heart disease, LDTD—lead-dependent tricuspid dysfunction, LVEF—left ventricular ejection fraction, MI—myocardial infarction, MRI—magnetic resonance imaging, SVC—superior vena cava, TLE—transvenous lead extraction.

**Table 2:** Patient-, system- and procedure-related risk factors for procedure complexity and major complications.

	No Stenosis 1	Mild Stenosis 2	Moderate Stenosis 3	Severe Stenosis 4	Total Occlusion 5	ANOVA Kruskal–Wallis Test (1–5) *p*	Mann–Whitney U/Chi^2^ Tests (1–2) vs. (4–5)
Number of Patients	N = 499 (17.15%)	N = 574 (19.73%)	N = 605 (20.80%)	N = 581 (19.97%)	N = 650 (22.34%)		
	Mean ± SD *n* (%)	Mean ± SD *n* (%)	Mean ± SD *n* (%)	Mean ± SD *n* (%)	Mean ± SD *n* (%)		
Patient-related risk factors for TLE complexity and complications							
Patient age during TLE [years]	64.48 ± 14.85	66.74 ± 13.69	66.64 ± 13.79	68.57 ± 13.04	67.68 ± 14.32	<0.001	<0.001
Male gender	325 (65.13)	324 (56.44)	324 (53.55)	342 (58.86)	447 (68.77)	<0.001	0.019
Etiology: IHD, MI	278 (55.71)	329 (57.31)	353 (58.34)	361 (62.13)	355 (54.62)	0.112	
Etiology: non-ischemic	221 (44.29)	245 (42.69)	252 (41.66)	220 (37.87)	295 (45.38)	0.112	
NYHA class III and IV (%)	67 (13.42)	96 (16.72)	71 (11.73)	196 (33.74)	99 (15.23)	0.523	
LVEF < 40%	158 (31.66)	173 (30.14)	177 (29.26)	196 (33.74)	215 (33.08)	0.847	
Renal failure (any)	88 (17.64)	119 (20.73)	122 (20.17)	121 (20.83)	156 (24.00)	0.211	
Previous sternotomy	81 (16.23)	85 (14.81)	79 (13.06)	76 (13.08)	114 (17.54)	0.210	
Charlson comorbidity index [number of points]	4.543 ± 3.789	4.702 ± 3.380	4.688 ± 3.589	5.086 ± 3.629	4.728 ± 3.550	0.038	0.016
**CIED system and history of pacing**							
Device type—pacemaker (any)	350 (70.14)	402 (70.04)	416 (68.76)	384 (66.09)	461 (70.92)	0.193	
Device type—ICD-V, ICD-D	126 (25.25)	127 (22.13)	144 (23.80)	141 (24.27)	127 (19.54)	0.142	
Device type—CRT-D	21 (4.208)	44 (7.666)	44 (7.237)	56 (9.639)	62 (9.54)	0.006	0.002
Redundant loop of the lead on X-Rays before TLE	20 (4.01)	21 (3.659)	25 (4.132)	26 (4.475)	44 (6.769)	0.074	0.047
Lead with proximal end in CVS before TLE	9 (1.804)	10 (1.742)	7 (1.150)	11 (1.893)	14 (2.154)	<0.001	0.762
Number of CIED-related procedures before TLE (SD)	1.728 ± 0.970	1.701 ± 0.881	1.777 ± 1.068	1.806 ± 0.959	2.088 ± 1.300	<0.001	<0.001
TLE before current TLE	23 (4.609)	23 (4.007)	19 (3.140)	27 (4.647)	39 (6.00)	0.200	
**Risk factors for major complications and procedure complexity**							
Number of extracted leads per patient [*n*]	1.597 ± 0.619	1.582 ± 0.681	1.623 ± 0.666	1.683 ± 0.719	1.835 ± 0.880	<0.001	<0.001
Three or more leads extracted	30 (6.012)	46 (8.014)	55 (9.090)	73 (12.57)	113 (17.38)	<0.001	<0.001
Approach: left	483 (96.79)	551 (95.99)	578 (95.54)	548 (94.32)	599 (92.15)	0.002	<0.001
Approach: right	7 (1.403)	9 (1.568)	12 (1.980)	17 (2.930)	11 (1.690)	0.443	
Approach: both	1 (0.200)	3 (0.523)	3 (0.496)	5 (0.860)	14 (2.154)	0.003	0.009
Approach: femoral	1 (0.200)	2 (0.348)	2 (0.331)	0 (0.00)	6 (0.923)	0.225	
Approach: subclavian-femoral	3 (0.601)	4 (0.697)	3 (0.496)	2 (0.344)	5 (0.970)	0.581	
Approach: other, combined	4 (0.802)	3 (0.523)	5 (0.826)	9 (1.549)	14 (2.150)	0.061	0.017
Approach: Jugular	0 (0.00)	2 (0.348)	0 (0.00)	0 (0.00)	0 (0.00)	0.086	0.420
Extraction of leads with redundant loop	14 (2.806)	14 (2.439)	20 (3.306)	21 (3.614)	37 (5.690)	0.026	0.011
Extraction of broken lead with proximal end in CS	11 (2.204)	11 (1.916)	7 (1.157)	10 (1.721)	16 (2.460)	0.756	
Extraction of abandoned lead(s) (any)	35 (7.014)	45 (7.840)	39 (6.446)	60 (10.33)	102 (15.69)	<0.001	<0.001
Extraction of abandoned lead(s) [*n*]	0.074 ± 0.277	0.103 ± 0.380	0.083 ± 0.340	0.138 ± 0.441	0.208 ± 0.520	<0.001	<0.001
Oldest extracted lead (months]	96.25 ± 73.71	102.4 ± 74.46	100.3 ± 76.37	94.21 ± 70.22	104.86 ± 76.70	0.078	
Average (per patient) extracted lead dwell time [months]	92.24 ± 67.47	97.85 ± 67.97	95.38 ± 70.31	88.59 ± 62.52	96.49 ± 67.68	0.163	
Cumulative dwell time of extracted leads [years]	12.30 ± 12.11	13.57 ± 12.43	13.55 ± 12.47	12.98 ± 11.59	15.39 ± 14.13	0 < 0.001	0.008
SAFeTY TLE calculator of risk for MC [points]	5.290 ± 4.117	5.828 ± 4.130	5.995 ± 4.249	5.597 ± 4.090	6.333 ± 4.560	0.002	<0.024
SAFeTY TLE calculator of risk for MC [%]	1.470 ± 2.566	1.621 ± 2.490	1.782 ± 3.430	1.608 ± 2.690	2.089 ± 3.650	0.002	<0.001

CRTD—implantable cardioverter-defibrillator with resynchronization function, CS—coronary sinus, ICD-D—dual chamber implantable cardioverter-defibrillator, ICD-V—single chamber implantable cardioverter-defibrillator, IHD—ischemic heart disease, LVEF—left ventricular ejection fraction, MI—myocardial infarction, NYHA—New York Heart Association functional class, TLE—transvenous lead extraction.

**Table 3:** TLE complexity.

	No Stenosis 1	Mild Stenosis 2	Moderate Stenosis 3	Severe Stenosis 4	Total Occlusion 5	ANOVA Kruskal–Wallis Test (1–5) *p*	ANOVA Kruskal–Wallis Test (1–2) vs. (4–5)
Number of Patients	N = 499	N = 574	N = 605	N = 581	N = 650		
	Mean ± SD *n* (%)	Mean ± SD *n* (%)	Mean ± SD *n* (%)	Mean ± SD *n* (%)	Mean ± SD *n* (%)		
TLE complexity							
Procedure duration (skin-to-skin) [minutes]	57.66 ± 22.54	59.18 ± 25.99	58.23 ± 20.90	59.33 ± 24.09	64.16 ± 33.85	0.018	0.075
Procedure duration (sheath-to-sheath) [minutes]	12.36 ± 19.06	13.26 ± 21.86	12.73 ± 17.14	13.56 ± 20.36	20.32 ± 32.45	<0.001	<0.001
Average time of single lead extraction [minutes]	8.149 ± 11.26	8.441 ± 15.05	7.755 ± 9.616	7.568 ± 8.884	10.56 ± 15.30	<0.001	0.126
All leads were extracted	399 (79.96)	432 (75.26)	450 (74.38)	420 (72.29)	503 (77.38)	0.040	0.181
Functional lead was left in place for continuous use	98 (19.64)	137 (23.87)	153 (25.29)	157 (27.02)	141 (21.69)	0.030	0.207
Non-functional lead was left in place	1 (0.200)	3 (0.523)	1 (0.165)	1 (0.172)	6 (0.923)	0.302	
Non-functional superfluous lead was extracted	35 (7.014)	45 (7.840)	39 (6.446)	60 (10.33)	102 (15.69)	<0.001	<0.001
Technical problem during TLE (any)	85 (17.03)	110 (19.16)	116 (19.17)	109 (18.76)	162 (24.92)	0.011	0.025
Block in implant vein (subclavian region)	34 (6.814)	39 (6.794)	48 (7.934)	43 (7.401)	65 (10.00)	0.154	
Lead-to-lead binding	28 (5.611)	33 (5.749)	39 (6.446)	42 (7.229)	64 (9.846)	0.030	0.009
Byrd dilator collapse/torsion/“fracture”	16 (3.206)	19 (3.310)	19 (3.140)	19 (3.270)	23 (3.538)	0.968	
Lead fracture during extraction	22 (4.409)	22 (3.833)	31 (5.124)	29 (4.991)	51 (7.846)	0.004	0.014
Need to change venous approach	12 (2.405)	13 (2.265)	14 (2.314)	18 (3.098)	41 (6.308)	<0.001	<0.002
Functional lead dislodgement	6 (1.202)	8 (1.394)	6 (0.992)	5 (0.861)	5 (0.769)	0.824	
Loss of lead fragment	2 (0.401)	8 (1.394)	6 (0.992)	5 (0.861)	5 (0.769)	0.800	
Reel of ICD lead coil	2 (0.401)	4 (0.697)	5 (0.826)	1 (0.172)	2 (0.308)	0.222	
Number of big technical problems	1.316 ± 0.637	1.325 ± 0.718	1.313 ± 0.685	1.330 ± 0.620	1.500 ± 0.784	0.002	0.053
One technical problem only	57 (11.42)	65 (11.32)	76 (12.56)	64 (11.01)	81 (12.46)	0.925	
Two technical problems	16 (3.206)	12 (2.091)	12 (1.983)	21 (3.614)	33 (5.077)	0.006	0.029
Three or more technical problems	3 (0.601)	6 (1.045)	8 (1.322)	3 (0.515)	14 (2.154)	<0.001	0.302
Other smaller technical problems	25 (5.010)	23 (4.007)	26 (4.298)	22 (3.787)	49 (7.538)	0.003	0.192
**Use of additional tools**							
Evolution (old and new) or tight rail	7 (1.403)	5 (0.871)	8 (1.322)	7 (1.205	17 (2.615)	0.121	
Metal sheath	30 (6.012)	36 (6.272)	50 (8.264)	40 (6.886)	63 (9.692)	0.064	0.051
Lasso catheter/snare	14 (2.806)	13 (2.265)	17 (2.810)	17 (2.926)	33 (5.077)	0.017	0.052
Basket catheter	7 (1.403)	6 (1.045)	4 (0.661)	2 (0.344)	8 (1.231)	0.284	

ICD—implantable cardioverter-defibrillator, TLE—transvenous lead extraction.

**Table 4:** TLE efficacy and complications, and long-term mortality after TLE.

	No Stenosis 1	Mild Stenosis 2	Moderate Stenosis 3	Severe Stenosis 4	Total Occlusion 5	ANOVA Kruskal–Wallis Test (1–5) *p*	ANOVA Kruskal–Wallis Test (1–2) vs. (4–5)
Number of Patients	N = 499	N = 574	N = 605	N = 581	N = 650		
	Mean ± SD *n* (%)	Mean ± SD *n* (%)	Mean ± SD *n* (%)	Mean ± SD *n* (%)	Mean ± SD *n* (%)		
TLE efficacy and complications							
Major complications (any)	8 (1.603)	10 (1.742)	11 (1.818)	16 (2.754)	16 (2.461)	0.599	
Hemopericardium	5 (1.002)	6 (1.045)	8 (1.322)	11 (1.893)	9 (1.385)	0.707	
Hemothorax	3 (0.601)	3 (0.523)	1 (0.165)	0 (0.00)	3 (0.462)	0.288	
Tricuspid valve damage during TLE	2 (0.401)	4 (0.697)	2 (0.331)	5 (0.861)	3 (0.462)	0.722	
Rescue cardiac surgery	4 (0.802)	3(0.523)	8 (1.322)	9 (1.549)	10 (1.538)	0.286	
Minor complications (any)	31 (6.212)	35 (6.098)	46 (7.438)	44 (7.229)	60 (8.615)	0.278	
Procedure-related death (intra-, post-procedural)	1 (0.200)	1 (0.174)	1 (0.165)	2 (0.344)	1 (0.154)	0.401	
Indication-related death (intra-, post-procedural	1 (0.200)	0 (0.00)	0 (0.00)	1 (0.172)	0 (0.00)	0.485	
Partial radiographic success (remained tip or <4 cm lead fragment)	13 (2.605)	18 (3.136)	22 (3.636)	18 (3.098)	34 (5.231)	0.203	
Complete clinical success	492 (98.60)	563 (98.08)	592 (97.85)	570 (98.11)	634 (97.54)	0.806	
Complete procedural success	481 (96.39)	550 (95.82)	578 (95.54)	556 (95.70)	611 (94.00)	0.322	
**Organizational model of TLE procedure. TEE monitoring**							
Routine TEE monitoring of lead extraction	261 (52.31)	266 (46.34)	263 (43.37)	263 (45.27)	233 (35.85)	<0.001	<0.001
**TLE-related TV dysfunction**							
Increase in TR by 1 degree	22 (4.409)	26 (4.530)	29 (4.793)	38 (6.540)	39 (6.000)	0.234	
Increase in TR by 2 degrees	8 (1.603)	5 (0.871)	13 (2.149)	10 (1.721)	11 (1.692)	0.632	
Increase in TR by 3 degrees	2 (0.401)	2 (0.348)	3 (0.496)	0 (0.00)	3 (0.462)	0.892	
Increase in TR by 2 degrees and to Grade IV	2 (0.401)	4 (0.697)	3 (0.496)	5 (0.861)	3 (0.462)	0.839	
Damage to chordae tendineae during TLE	14 (2.806)	15 (2.613)	18 (2.975)	24 (4.131)	25 (3.846)	0.531	
**Short-, mid- and long-term mortality after TLE**							
Survival in 1712 ± 1187 (1–4638) days of follow up	375 (75.15)	389 (67.77)	436 (72.07)	373 (64.20)	409 (62.92)	<0.001	<0.001
Death within 48 h	3/499 (0.601)	1/574 (0.174)	1/605 (0.165)	3/581 (0.516)	2/650 (0.308)	0.794	
One-month mortality; 2–30 days; *n* (% of patients with follow-up longer than 2 days)	4/495 (0.808)	9/573 (1.571)	5/604 (0.828)	4/578 (0.692)	7/648 (1.080)	0.464	
One-year mortality (31–365 days); *n* (% of patients with follow-up >30 days)	31/491 (6.314)	40/558 (7.168)	43/597 (7.203)	38/573 (6.632)	37/640 (5.781)	0.889	
Three-year mortality (366–1095 days); *n* (% of patients with follow-up >365 days	24/433 (5.543)	51/482 (10.581)	50/514 (9.728)	74/499 (14.83)	59/579 (10.19)	<0.001	<0.001
Death at >3 years (at 1095 days); *n* (% of patients with follow-up >1095 days)	62/359 (17.27)	84/429 (19.58)	70/447 (18.57)	89/447 (19.91)	136/569 (23.90)	<0.001	<0.001

TEE—transesophageal echocardiography, TLE—transvenous lead extraction TR—tricuspid valve regurgitation, TV—tricuspid valve.

## Data Availability

Readers can access the data supporting the conclusions of the study at www.usuwanieelektrod.pl (accessed on 11 September 2021).

## Abbreviations

AnV	innominate (brachiocephalic) vein
AxV	axillary vein
CIED	cardiac implantable electronic device
CRT	cardiac resynchronization therapy
EF	ejection fraction
FU	follow-up
ICD	implantable cardioverter-defibrillator
IVC	inferior vena cava
LR	lead-related
LRVSO	lead-related venous stenosis/occlusion
LV	left ventricle
LVEF	left ventricular ejection fraction
NYHA	The New York Heart Association (functional class)
Pts	patients
PM	pacemaker
RA	right atrium
RV	right ventricle
TEE	transesophageal echocardiography
TLE	transvenous lead extraction
ScV	subclavian vein
SVC	superior vena cava
TV	tricuspid valve
VSO	venous stenosis/occlusion
